# DECODE: a computational pipeline to discover T cell receptor binding rules

**DOI:** 10.1093/bioinformatics/btac257

**Published:** 2022-06-27

**Authors:** Iliana Papadopoulou, An-Phi Nguyen, Anna Weber, María Rodríguez Martínez

**Affiliations:** IBM Research Europe, 8803 Rüschlikon, Switzerland; ETH Zurich, Department of Biosystems Science and Engineering (D-BSSE), 4058 Basel, Switzerland; IBM Research Europe, 8803 Rüschlikon, Switzerland; ETH Zurich, Department of Mathematics (D-Math), 8092 Zurich, Switzerland; IBM Research Europe, 8803 Rüschlikon, Switzerland; ETH Zurich, Department of Biosystems Science and Engineering (D-BSSE), 4058 Basel, Switzerland; IBM Research Europe, 8803 Rüschlikon, Switzerland

## Abstract

**Motivation:**

Understanding the mechanisms underlying T cell receptor (TCR) binding is of fundamental importance to understanding adaptive immune responses. A better understanding of the biochemical rules governing TCR binding can be used, e.g. to guide the design of more powerful and safer T cell-based therapies. Advances in repertoire sequencing technologies have made available millions of TCR sequences. Data abundance has, in turn, fueled the development of many computational models to predict the binding properties of TCRs from their sequences. Unfortunately, while many of these works have made great strides toward predicting TCR specificity using machine learning, the black-box nature of these models has resulted in a limited understanding of the rules that govern the binding of a TCR and an epitope.

**Results:**

We present an *easy-to-use* and *customizable* computational pipeline, DECODE, to extract the binding rules from *any* black-box model designed to predict the TCR-epitope binding. DECODE offers a range of analytical and visualization tools to guide the user in the extraction of such rules. We demonstrate our pipeline on a recently published TCR-binding prediction model, TITAN, and show how to use the provided metrics to assess the quality of the computed rules. In conclusion, DECODE can lead to a better understanding of the sequence motifs that underlie TCR binding. Our pipeline can facilitate the investigation of current immunotherapeutic challenges, such as cross-reactive events due to off-target TCR binding.

**Availability and implementation:**

Code is available publicly at https://github.com/phineasng/DECODE.

**Supplementary information:**

Supplementary data are available at *Bioinformatics* online.

## 1 Introduction

The adaptive immune system relies on the selective recognition of foreign antigens by T cells (Kumar *et al.*, 2018). T cells recognize short antigenic peptides presented on major histocompatibility complex (MHC) proteins by binding the complex with their T cell receptors (TCRs). A large diversity of TCRs (Laydon *et al.*, 2015) ensures that all of the vast space of possible antigenic peptides can be recognized by the immune system.

In recent years, experimental advances have allowed the identification of T cells that recognize specific antigens. High-throughput sequencing of their TCRs has allowed linking large numbers of TCR sequences to antigen specificity. The increase in available data regarding TCR specificity has resulted in the development of many machine-learning models aiming to predict TCR specificity from sequences (Dash *et al.*, 2017; De Neuter *et al.*, 2018; Fischer *et al.*, 2020; Gielis *et al.*, 2019; Glanville *et al.*, 2017; Jokinen *et al.*, 2021; Moris *et al.*, 2021; Sidhom *et al.*, 2021; Weber *et al.*, 2021). These efforts are paving the way for the potential decoding of the information present in a patient’s TCR repertoire regarding its immune history, which might reveal information about past and present infections, as well as about disorders of the immune system, such as autoimmune diseases.

Unfortunately, TCR specificity prediction models are often black-box algorithms with *limited interpretability*. That means that despite the continuous improvement in predictive performances, these models fail to provide insights about the biochemical rules that govern the TCR–epitope interaction. Understanding and being able to accurately predict the binding of new TCRs to target epitopes is an essential step to design more powerful and safer T cell therapies, which represent a promising new approach for cancer immunotherapies.

Motivated by the need of interpretation in the TCR-epitope binding prediction problem, in this work, we provide an *easy-to-use* explainability pipeline to extract biological insights from *any* TCR-binding prediction model.

### 1.1 Motivation

In order to increase the explainability of machine-learning models, recent work, e.g. Tcr epITope bimodal Attention Networks (TITAN) (Weber *et al.*, 2021), has proposed to augment deep-learning architectures with interpretable components, such as attention layers (Vaswani *et al.*, 2017). This is an example of *ante-hoc*, or built-in, strategies to tackle interpretability. However, the application of attention mechanisms and, more generally, *ante-hoc* approaches to the TCR-binding prediction problem suffers from two main drawbacks:

- *Local interpretability*: given the complex nature of both the model and the task, *ante-hoc* techniques often generate only local explanations, i.e. explanations that are valid only for few samples. This results at best in the *partial* understanding of the biological mechanism underlying TCR binding.
- *Modification and retraining of the original model is needed*: *ante-hoc* approaches need to be embedded in the existing models, which requires retraining the whole new model *from scratch*. Disregarding the amount of work that this might require, this is only possible if the model’s code has been released. Furthermore, there is no guarantee that the modification does not result in a decrease in performance (Fischer *et al.*, 2020).

To address these issues, we introduce DECODE (DEcoding t Cell receptOr binDing rulEs), a *post-hoc* explainability pipeline that does not need access to a model’s architecture, and therefore, can be applied to models with proprietary or unreleased code. DECODE has been designed to discover the rules that *any* TCR specificity prediction algorithm follows to make its predictions. Our only requirement is to have access to a black-box model that can make a binding/non-binding prediction for a given TCR-epitope pair.

The two main components of DECODE are:

- A *clustering* step to enable *global* explainability. This allows the user to understand a model in its entirety and not only on individual cases.
- An *explainability* step based on Anchors, a model-agnostic approach that explains the behavior of complex models using high-precision rules (Ribeiro *et al.*, 2018).

Using these two components, DECODE creates explanations for *each* cluster of TCRs in the form of easily understood *if-then* rules.

DECODE is easy to use, offers a wide range of visualizations for the generated binding rules and can be applied to any text/categorically based model. We demonstrate our pipeline on the recently published TITAN model (Weber *et al.*, 2021). To facilitate the visualization of the generated explanations and the investigation of the associated TCR-binding mechanisms, we have retrained TITAN to predict the binding to a single-epitope, the peptide KLGGALQAK from Cytomegalovirus.

### 1.2 Related work

Several works have recently focused on predicting TCR specificity from sequence using machine learning [for a review see Mösch *et al.* (2019)]. Since the small number of epitopes is currently a limiting factor, many approaches have limited their scope and build supervised classifiers that predict the binding of a TCR to a limited pool of epitopes. Among these models, decision trees (De Neuter *et al.*, 2018; Gielis *et al.*, 2019) and Gaussian process methods (Jokinen *et al.*, 2021) have been proposed. On the other side, TITAN (Weber *et al.*, 2021) used a bimodal, sequence-based neural network for predicting TCR-epitope binding probability and significantly outperformed the state-of-the-art.

Regarding the problem of identifying informative motifs and sequence patterns that confer TCR its specificity properties, several approaches have been proposed. GLIPH (Glanville *et al.*, 2017) clusters TCRs according to amino -acid similarity and the presence of conserved motifs; TCR-specific distance measures have been defined to enable clustering and visualization of similar TCR sequences (Dash *et al.*, 2017); and variational autoencoders have been used to improve the extraction of TCR meaningful features, named concepts, from TCR repertoires (Sidhom *et al.*, 2021).

With the exception of TITAN, which uses attention layers to highlight informative amino acids, none of the previous approaches has focused on investigating the biochemical rules that govern TCR binding. As discussed in Section 1.1, attention mechanisms can highlight patterns in the input data that a neural network finds informative; however, they cannot provide human-understandable rules predictive of TCR binding.

Here, we aim to explore alternative approaches for interpretability. Besides *ante-hoc* approaches to interpretability, i.e. models that are interpretable by construction, such as decision trees, many *post-hoc* approaches have been developed in recent years. These methods can shed light on the decision process of a machine-learning model after it has been trained. They are often model-agnostic, in the sense that they do not need any information about the model itself, and are therefore applicable to any predictive model. Two popular examples are LIME (Ribeiro *et al.*, 2016), which learns a local linear model, and Anchors (Ribeiro *et al.*, 2018), which generates local *if-then* rules, similar to a local decision tree. Feature attribution methods are another type of *post-hoc* interpretable methods that aim to highlight the features that are most important toward a prediction. However, while they can detect *which* features may have been used for a prediction, they do not elucidate *how* they are used. Feature attributions can be either model specific, e.g. gradient-based methods (Ancona *et al.*, 2018) applied to a neural network, or model agnostic, e.g. SHAP (Lundberg and Lee, 2017).

In this work, we choose Anchors as the core component of the explainability step of DECODE. Anchors does not makes any linearity assumption (as LIME does), and therefore is able to find more complex rules. Further, logical rules can be more directly interpreted by human experts (Fürnkranz *et al.*, 2020) compared to importance scores, and thus Anchors are expected to result in more user-friendly explanations than feature attribution methods.

## 2 Materials and methods

### 2.1 Overview of the pipeline

**Fig. 1.**
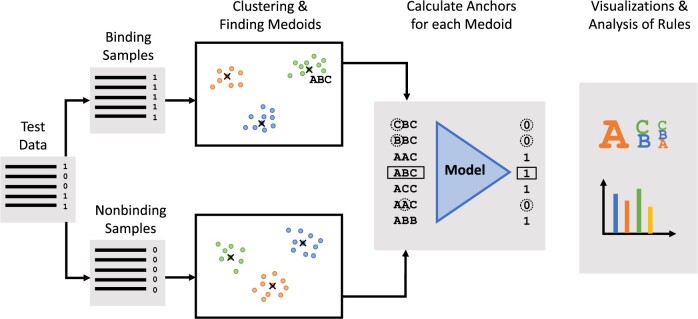
Overview of DECODE interpretability pipeline. The test data are split into binding and non-binding samples based on the predictions of the model. Both sets of samples are clustered and the medoid of each cluster is chosen as a representative example of the cluster. Note that the medoid is an actual sample from the cluster it represents. The pipeline allows for the use of different clustering algorithms, see Section 2.3. For each medoid, DECODE generates a set of rules using Anchors. Finally, the pipeline offers a range of visualizations and evaluation metrics for the final anchor rules

In this section, we expand on the computational steps of the DECODE pipeline. Figure 1 graphically shows the main steps, consisting of:

1. A preparation phase (Section 2.2) to load the model to interpret and to prepare the data;
2. A clustering phase (Section 2.3), where representative samples of the dataset heterogeneity are identified;
3. An explainability phase (Section 2.4), where the explanations about the representative samples retrieved at the previous step are computed.

Our pipeline further provides a framework to analyze and evaluate both the clustering (Section 2.3.1) and the generated explanations (Section 2.4.1). The user can define the specific algorithms to use in the preprocessing and clustering phase, as well as other parameters, in an *easily customizable*json file. For the simplest predictive models, the user does not need to inspect the code and can setup the pipeline simply by specifying the mentioned configuration json file, making the pipeline particularly user-friendly even for practitioners *with little to no experience in programming*.

### 2.2 TCR prediction model and data preparation

The very first step of our pipeline takes care of loading the model and the data. The model should provide a predict function that returns a predicted class for an input batch. The data should be loaded in a format compatible with the model. For the simplest cases [e.g. a scikit (Pedregosa *et al.*, 2011) model using data in a numpy (Harris *et al.*, 2020) array format], our pipeline already provides the loading functions. For more complex cases [e.g. tensorflow (Abadi *et al.*, 2015), pytorch (Paszke *et al.*, 2019) or command-line tools], the user should provide their own loading functions.

After loading, the pipeline will take care of splitting the samples in binding and non-binding partitions, as predicted by the loaded model. In the remainder, we will refer to these partitions as *splits*. The clustering will be run *separately* for these two partitions. Since we expect the binding and non-binding rules to be different, this separation step will help the explainability phase to detect ‘higher quality’ rules.

As the final (sub)step of this first phase, the user can optionally specify how to further (pre)process the data. This step is necessary only if the data format expected by the model is not the same as the format expected by the clustering and explainability steps. For example, the user may want to provide a precomputed distance matrix to speed up some clustering algorithms.

### 2.3 Clustering

After the loading and preparation phase, the pipeline will identify clusters in each of the samples splits. The pipeline readily provides different clustering algorithms, including K-medoids (Park and Jun, 2009), BIRCH (Zhang *et al.*, 1996) or OPTICS (Ankerst *et al.*, 1999). Similarly to the loading functions in the previous step, the user can provide their own custom clustering algorithm.

In case it is not clear which clustering algorithm (or which hyperparameters) should be preferred, our pipeline allows the user to run multiple algorithms. The pipeline then provides facilities to select the best clustering algorithm according to a user-defined criterion (Section 2.3.1).

Furthermore, if different clustering outcomes are produced, the pipeline offers the possibility of finding a consensus clustering using the ClusterEnsemble package (Strehl and Ghosh, 2003).

#### 2.3.1 Clustering evaluation

The pipeline provides both quantitative and qualitative ways to evaluate the clustering algorithms (and the hyperparameters) selected by the user. Quantitative methods include established criteria, such as the davies-bouldin score (Davies and Bouldin, 1979) or the Silhouette coefficient (Rousseeuw, 1987). The qualitative assessment strategies consist of dimensionality reduction algorithms that will project the samples to a 2D space and color-code them using the labels provided by each clustering algorithm. Examples of provided projection methods are t-SNE (Van Der Maaten and Hinton, 2008) and LLE (Locally Linear Embeddings) (Roweis and Saul, 2000). We show an example of clustering evaluation and visualization in Section 3.3.

### 2.4 Anchors

We generate human-readable explanations using Anchors (Ribeiro *et al.*, 2018). An anchor is an explanation presented as an *if-then* rule. More specifically to our use case, *for each of the cluster representative samples*, the computed rule takes the following form:

IF (amino acid X is in position i)

 
 
 
AND (amino acid Y is in position j)

 
 
 
AND etc.. THEN

the samples is PREDICTION,

where X and Y represent some amino acid, *i* and *j* may take any value within the length of the input and PREDICTION depends on what kind of rule we are trying to explain (i.e. either *binding* or *non-binding*).

The *quality* of these rules and the *completeness* of the overview they provide in explaining a TCR prediction model depend on a number of factors, including the clustering algorithm and the hyperparameters of the Anchors algorithm (discussed in Section 3.4). In the next section, we introduce some metrics to evaluate anchors.

Finally, note that there exists a trade-off between the quality of the explanations and the computational resources (memory and time) required for their generation. For reference, in our experiments on a single Intel Xeon CPU E5-2667 (@3.30 GHz), computing a single anchor with default parameters requires between 15 min and 3 h, depending on the complexity of the rule. With less restrictive parameters leading to lower quality rules, the computation can be shortened to 2 min up to 30 min.

#### 2.4.1 Anchors evaluation

Ideally, we would like a set of anchors to *faithfully* represent the underlying model and be *complete* with respect to the whole space of samples. In layman terms, this means that:

- The anchors should be able to replicate the same prediction of the model. That means that if a sample fulfills an anchor rule, this rule should lead to the same prediction as the one provided by the model.
- The anchors should not *overlap*, i.e. different anchors should not be applicable to the same sample simultaneously. This is especially important if the rules were generated from clusters belonging to different data splits (Section 2.2). If a sample fulfills two anchor rules associated with different predictions, how can a user decide which one is correct?
- The set of anchor rules should be able to cover the whole sample space. Only if this condition is fulfilled, we may be able to understand globally the model.

**Fig. 2.**
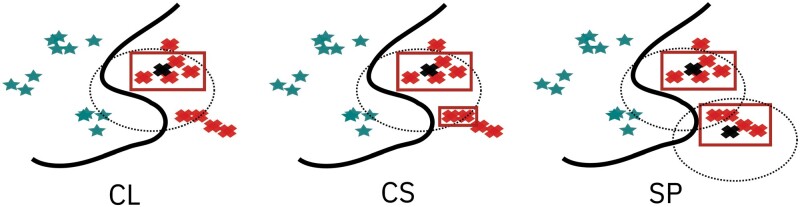
Cluster metrics: graphical visualization. Blue stars represent samples predicted as binding by the model we want to explain. Red crosses are samples predicted as non-binding. The thick continuous line is the decision boundary of the model. We can identify three clusters for the binding samples, and two for the non-binding ones. As an example, we graphically visualize the three levels of metrics applied to the non-binding anchors. The black crosses denote the medoids of the (non-binding) clusters. Dotted circles denote the boundary decisions of the computed anchors: all the samples within the circle fulfill the anchor rule. Red rectangles highlight the true positives according to the anchor rules. CL metrics considers true positives the samples that fulfill the anchor rule *and* belong to the same cluster as the medoid used to compute the rule. CS level metrics considers true positives all the samples that fulfill the anchor rule and belong to the *same split* as the medoid considered. Finally, SL metrics considers true positives all the samples that fulfill any of the anchor rules of a split and belongs to that same split

The last two points mean in particular that any admissible input sample should ideally fulfill exactly one of the generated anchor rules. The listed desiderata can be evaluated using standard predictive performance metrics (*accuracy*, *precision* and *recall*), and other overlap/completeness metrics that we will introduce later in this section. The predictive performance metrics follow the usual definitions:

$$\begin{array}{c} \text{Accuracy}(A)=\frac{TP+TN}{TP+TN+FP+FN}\\ \text{Precision}(P)=\frac{TP}{TP+FP}\\ \text{Recall}(R)=\frac{TP}{TP+FN}, \end{array}$$

where TP are true positives, TN are true negatives, FP are false positives and FN are true negatives. We provide these metrics at three different levels, and the definition of a TP will depend on the level at which the metric is computed. For a visual description of these metrics, please refer to Figure 2. A more detailed description of the figure can be found in the Supplementary Section S1.

- **Cluster level** (CL): a sample is considered a true positive if it fulfills *only* the anchor rule generated from the medoid of its cluster. Perfect performance at this level (simultaneously for *all* the clusters) would give us all the desiderata mentioned above. In particular, if all anchor rules are accurate, then they are faithful; if they are all precise, then there is no overlap; and if they all have perfect recall, then we have complete coverage.
- **Cluster-split** **(CS)** **level** : an anchor rule prediction on a sample is considered a true positive if it leads to the same model-prediction of the sample. In particular, the sample does not need to belong to the same cluster from which the anchor is generated. In turn, this means that high precision does not guarantee *no-overlap*. This level is more relaxed than the previous one: low performance at the CL level, but high performance on the CS level may be acceptable. This would mean that there are overlapping rules, but at least these rules lead to the same prediction. However, while this situation might be acceptable from a predictive point of view, it poses a problem for interpretability. For instance, if two rules apply, which one should we choose? Should both the rules be considered simultaneously?
- **Split level** (SP): a sample is considered a true positive if it fulfills *any* of the rules generated from the clusters of its same *split*. This is a further relaxation of the CL level.

A different way of understanding the differences between the different anchor metrics is by considering the question being answered by each metric. At the CL, we are answering the question how many samples in a particular cluster fulfil the cluster's rule. This leads to a score per cluster. At the CS level, we are answering the question “how many samples in a split fulfil the cluster's rule”. The answer also leads to a score per cluster. At the SP level, the question being answered is “how many samples in a split fulfil any of the rules”. Therefore, we only get one score for the whole set of anchor rules (see Figure 4 for an example).

If a set of anchors performs poorly on the above metrics, we might conclude that this set is not ideal, in terms of either faithfulness or completeness. Computing the overlap and completeness of the rules might result in useful information to adjust the parameters of the pipeline. With this goal in mind, we propose a simple counting measure to monitor the degree of overlap between rules. Given two anchors, the *overlap* is the number of (sub-)rules that are compatible, i.e. they require the *same* amino acids in the *same* positions. For anchors computed from the same split, it is acceptable to have a high count of overlaps. This might simply mean that the rules are redundant. However, high overlap is not desirable for anchors generated from different splits: if both rules apply and lead to conflicting predictions, how should the sample be interpreted?

To monitor the degree of completeness, for each sample in a validation/test set, we simply count the number of anchors that are applicable to that sample. Ideally, all samples should fulfill one and only one anchor. If there are a high number of samples with zero applicable anchors, it would mean that the generated set of anchors is far from completeness.

#### 2.4.2 Anchors visualization

To facilitate interpretability, we visualize the textual anchor rules similarly to a sequence motif (e.g. Fig. 7). Since models may take as an input very long sequences, we further facilitate rule visualization by allowing the split of the motif in multiple regions, e.g. the Framework Regions (FRs) and Complementarity-Determining Regions (CDRs) for a TCR.

## 3 Results

As a proof of concept, we apply our pipeline to two recently published deep-learning models for TCR-binding prediction, TITAN (Weber *et al.*, 2021) and pMHC-TCR-binding prediction network (pMTnet) (Lu *et al.*, 2021). To keep our proof of concept simple, we analyze both models only on a *single epitope*: the peptide KLGGALQAK from Cytomegalovirus. This peptide was selected for being the one with the largest number of associated TCRs in our dataset (Section 3.2).

### 3.1 The models to interpret

#### 3.1.1 TITAN

TITAN (Weber *et al.*, 2021) is a bimodal attention-based neural network that takes as input a sequence representing an epitope and a sequence representing a TCR.

TITAN can leverage different encodings, but in our experiments, we use a SMILES (Weininger, 1988) representation for the fixed epitope and BLOSUM encodings for the TCRs. Both sequences are padded with a special ‘<PAD>’ token so that the lengths of the input sequences for TITAN are fixed to 500 for the epitope and 500 for the TCR. To help the training of the model, the actual TCR sequence is preceded by a ‘<START>’ token and is followed by a ‘<STOP>’ token. We specifically retrain TITAN for 50 epochs on a dataset containing solely the selected epitope. Details about the dataset are provided in the next section. On this dataset, TITAN achieves an accuracy of 77%.

#### 3.1.2 pMTnet

pMTnet (Lu *et al.*, 2021) is a transfer learning-based model that predicts TCR -epitope binding based on an Atchley factor encoding of the *CDR3* loop of the TCR and a joint encoding of epitope and MHC similar to the netMHCpan model. pMTnet outputs the fractional rank of each TCR compared to 10 000 background receptors, i.e. its rank divided by 10 000, which we use as a proxy for the binding probability. We load the trained model provided on Github and interpret the predictions on our test set, where the model achieves an accuracy of 50%. We want to highlight that our test dataset is not well suited for a fair judgment of pMTnets performance, since it likely contains more noise than the dataset that pMTnet was trained to achieve high performance on and we did not retrain or finetune pMTnet on the single -epitope prediction task.

### 3.2 Data

As mentioned above, we choose to focus only on the peptide KLGGALQAK. We build our peptide-specific dataset starting from a 10X Genomics study (10X Genomics, 2020), which identified 13 647 different TCRs binding to this peptide. Since no true negative data exists, we create non-binding pairs by retrieving TCRs that have not been found to bind to this peptide. We compose this ‘negative’ subset using three different sources:

- 4207 TCRs included in the VDJ database (Bagaev *et al.*, 2020) that bind to different targets;
- 3951 naive TCR sequences from another 10X Genomics study; and
- 5399 TCR sequences generated with the tool IGor (Marcou *et al.*, 2018).

For our analysis, we focus solely on TCR*β* chain sequences. After processing and cleaning, we obtain a balanced dataset of 21 832 samples, which we split in a training/test set. We run the interpretability analysis for both models on the *same* test set comprised of 2K samples.

### 3.3 Clustering results

As underlying distance metric for the clustering phase, we use the Levenshtein distance, a string metric that counts the number of amino-acid edits (insertions, deletions or substitutions) necessary to turn a sequence into another. Unfortunately, not all algorithms allow for arbitrary distances. Therefore, in our case study, we restrict our analysis to K-Medoids, OPTICS and AgglomerativeClustering (Müllner, 2011). We run K-Medoids and AgglomerativeClustering with the number of clusters varying in a range between 5 and 20. For OPTICS, we define a range for the density parameter *ϵ* that leads to a comparable number of clusters (between 2 and 31).

**Fig. 3.**
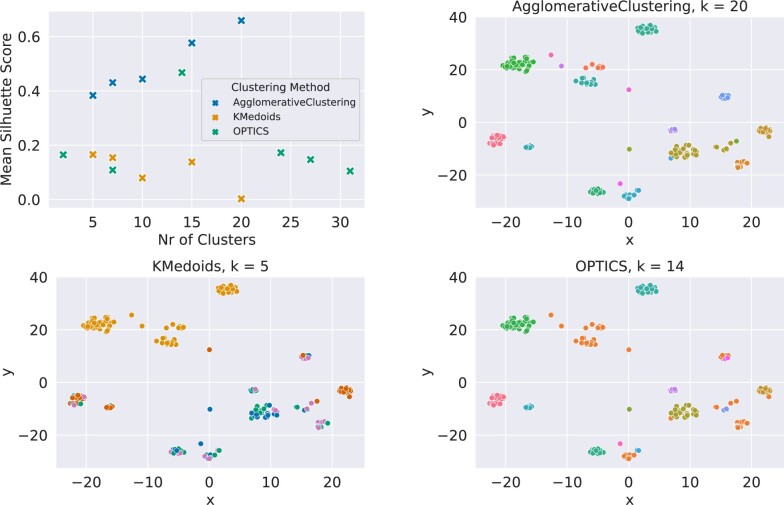
Comparison of clustering algorithms. Top-left shows the mean Silhouette score *s* for all three different clustering methods and a number of different numbers of clusters. AgglomerativeClustering achieves high scores for all numbers of clusters, with higher scores for higher numbers of clusters and a maximum of *s *=* *0.66 for *k *=* *20. OPTICS shows an optimum for *k *=* *14 clusters with a score of *s *=* *0.47, while *K*-medoids has low scores for any number of clusters. The other plots show t-SNE plots, where samples are color-coded according to their cluster assignment. The plots were generated for the number of clusters resulting in the highest Silhouette score for each clustering method, respectively. All clustering results are shown for the binding class

Because of the same distance metric constraint as above, we are restricted to use the Silhouette score for the quantitative assessment of the cluster partitions. For qualitative analysis, we use t-SNE. Figure 3 shows the Silhouette score for each algorithms and different number of clusters. For each algorithm, we further plot the t-SNE projections color-coded according to the best clustering as measured by the Silhouette score, e.g. for AgglomerativeClustering, we use the cluster labeling obtained by setting the number of clusters to 20.

For demonstration purposes, we focus this part of the analysis on the binding split. However, similar results are obtained for the non-binding split (see Supplementary Section S2).

From Figure 3, we notice that the top performing clustering for the binding split is AgglomerativeClustering with 20 clusters. Note that clustering metrics are just a *heuristic* to guide the selection of the clustering algorithm, but there is no guarantee that this will lead to the best possible anchor explanations. This selection step is necessary since the algorithm to compute Anchors is computationally expensive, and running it for each possible clustering result may be prohibitive.

We further note that the best OPTICS setting (ϵ=0.5, corresponding to 14 clusters) seems to be qualitatively similar to AgglomerativeClustering with 20 clusters, as per t-SNE visualization. As we mentioned above, quantitative clustering metrics may not correlate with the final quality of anchors. Therefore, a user may decide to select OPTICS over AgglomerativeClustering in order to compute a lower number of anchor rules, and reduce the computational burden. In the following, we select the results from the Agglomerative Clustering algorithm as a basis for the explainability phase.

### 3.4 Anchors metrics

**Fig. 4.**
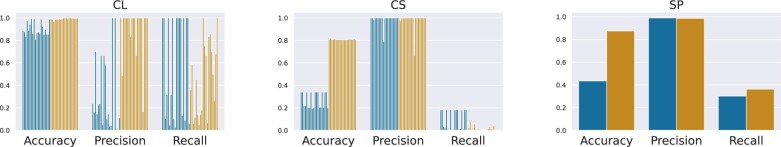
Anchors metrics. The accuracy, precision and recall of non-binding anchors (blue) and binding anchors (orange) are shown for the anchors generated from the clusters identified by the AgglomerativeClustering algorithm with 20 clusters. From left to right: CL metrics, CS level metrics and SP metrics for a total of 40 anchors (20 non-binding, 20 binding). Note that for the split metrics, we are jointly considering the 20 rules for each split at the same time (resulting in one metric per split)

The pipeline runs the Anchors algorithm as provided by the alibi package (Klaise *et al.*, 2021) with parameters that generate more precise rules compared to the default parameters. More precisely, we use δ=0.3, τ=0.3 and a threshold of 0.9. Figure 4 shows the metrics at the three different levels, as introduced in Section 2.4.1.

The leftmost plot in Figure 4 shows the metrics at the CL. Overall, the non-binding anchors (blue) and binding anchors (orange) seem to do a good job at identifying their own cluster (relatively high-accuracy). Notable difference is the fact that, tendentially, non-binding anchors have low precision and high recall, while the contrary holds true for binding anchors. Simply put, the Anchors algorithms generate for non-binding clusters decision rules that are less-specific for their own clusters. On the other hand, the rules generated for binding clusters seem to be too specific to the cluster centroid. That is, the binding anchor rules do not seem to well represent the other samples belonging to their own cluster.

Since non-binding anchors perform reasonably well at retrieving samples from their own cluster (high recall), a legitimate question is: how general are the non-binding rules? That is, do non-binding anchors recognize also non-binding samples not belonging to the same cluster? The CS level metrics (center plot in Fig. 4) help us answer these questions. Interestingly, now the non-binding anchors behavior is more similar to the binding ones. This means that the validity of a non-binding rule extends solely to its cluster, and not much further. Unsurprisingly, the specificity of the binding rules is further exacerbated at the SL.

The SL metrics help us understand if the rules belonging to the same split may *collectively* be able to identify their own split. The rightmost plot in Figure 4 shows that the set of rules retains similar accuracy/precision performance to the CS level, while significantly improving on the recall. This means that overall the Anchors algorithm managed to compute explanations that are faithful to the underlying biology, but not complete/global enough. That is, we have gained an understanding of how the model is functioning only for a subset of samples.

In summary, these multi-level metrics help us understand if the explanations generated from the pipeline are faithful to the model, and the extent of their validity/globality. They further may help us pinpoint which steps of the pipeline may require improvement. For example, changing the default Anchors parameters may improve the generality of binding rules (higher recall). Further, the ability of non-binding rules to recognize their own cluster, while not being able to collectively recognize the non-binding split, suggests that a different clustering algorithm/parameters setting could be used to find better representative non-binding samples.

**Fig. 5.**
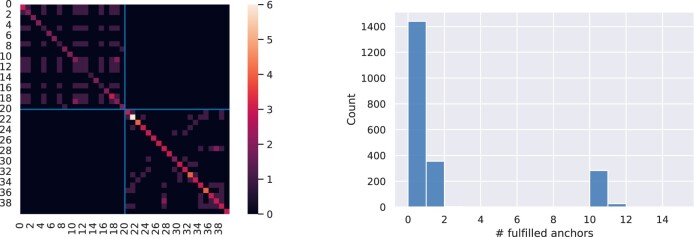
Anchors overlap and completeness analysis. Results are reported for the test set consisting of around 2k samples. Left plot shows the overlap between anchors visualized as a heatpmap: non-binding rules are located in top-left corner of the matrix, while binding rules in the bottom right. Color denotes the number of overlapping rules, with brighter colors indicating a higher number of rules. While there is some overlap of anchors rules within the same data splits (i.e. the binding and non-binding partitions), there is no overlap across splits. That means that no sample fulfills both a binding and a non-binding rule. Right plot shows completeness, i.e. a histogram depicting how many samples (*y*-axis) fulfill a certain number of anchors (*x*-axis). With the pipeline settings used for this experiment, most samples do not fulfill any anchor rule, around 300 samples fulfill either 1 or 10 rules, and few fulfill 11 rules. Therefore, the rules obtained in this experiment are not a *complete* set of rules

To have a clearer idea of how to change the pipeline settings to improve the generated explanations, we can have a look at the rule overlaps and the completeness/coverage of the rules. The overlaps between anchors can be visualized as a heatmap, similarly to interaction matrices (left plot in Fig. 5). The completeness can be readily visualized with a histogram (right plot in Fig. 5) counting how many (*y*-axis) samples fulfill a certain number of anchors (*x*-axis).

The left plot in Figure 5 suggests that there is quite some overlap between non-binding rules (top-left quadrant of the interaction matrix), which together with the previous conclusions, may suggest that a solution is to select a different clustering algorithm that *better separates* the clusters. As desirable, there is no overlap between non-binding and binding rules.

The anchor fulfillment histogram reveals that there is a considerably high number of samples not covered by the anchors, in agreement with the SL metrics. Namely, most samples do not fulfill any anchor rule, around 300 samples fulfill 1 rule and 10 rules, and a few samples fulfill 11 rules. The latter samples are likely to be non-binding samples whose rules tend to be less specific. This visualization confirms once again that a better clustering algorithm (or better parameters/metrics) might be the right solution.

An ablation study (Supplementary Section S3) confirms that by varying clustering parameters, we can trade-off precision, coverage and completeness of the generated anchor rules.

### 3.5 Anchors visual analysis

While the perfomance metrics shown in the previous section are far from ideal, this does not mean that we cannot attempt a biological interpretation of the model. While the previous results suggest that the explanations may not be able to give a complete overview of the model, they do seem to provide a correct interpretation.

#### 3.5.1 Distribution of rules in biologically significant regions

**Fig. 6.**
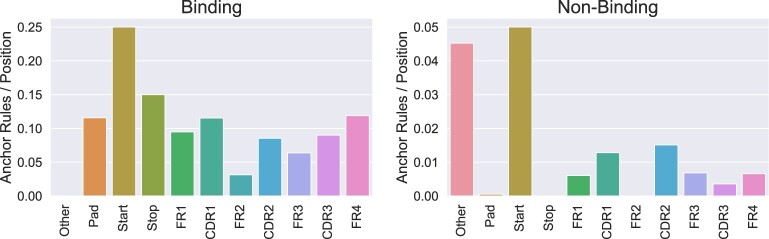
Histograms showing the distribution of anchor rules on the different regions of a TCR sequences normalized to the length of the region. The figure reports the number of anchors on the four FRs, three CDRs, other regions of the sequence not aligned with either the FRs or the CDRs (‘Other’), in the padded regions (‘PAD’), and on either the start or stop token (‘Start’/‘Stop’). Left plot: binding rules. Right plot: non-binding rules

The input sequences for an underlying TCR-binding prediction model may be too long for the user to properly interpret them. Indeed, in our case study, the input TCR sequences are 150 (500 if we consider the padding) characters long (Section 3.1.1). To make the anchor analysis more user-friendly, we provide functionalities to align samples and anchor rules, and extract various regions of interest, i.e. the FRs and CDRs (Section 2.4.1). A preliminary overview of the locations of anchor (sub)rules can be obtained by counting how many anchor rules fall into each of the regions. For a more fair comparison of the regions, these counts are normalized by the length of each of the regions. Figure 6 reports these counts separately for non-binding and binding splits. Apart from the four FRs and three CDRs, we report counts also for rules falling in a part of the sequence that was not aligned to either the FRs or the CDRs (‘Other’), in the padded region of the sequence (‘PAD’), or on either the start or stop token (‘Start’/‘Stop’).

Interestingly, both non-binding and binding rules seem to put a similar emphasis on the CDR2 and CDR1 regions as on the CDR3. While it is generally agreed that the CDR3 region is more directly implicated in binding an epitope, which might lead to an expected higher concentration of rules in this region, all CDRs are physically involved in the binding, and therefore, it is reasonable to find rules concentrating on the CDR2 and CDR1 region. Another interesting result is the fact that TITAN binding rules seem to have a preference also for the FRs. FRs have a supporting role for binding, e.g. by increasing the surface density of TCRs, without impacting their affinity (Thomas *et al.*, 2019). Rules in FRs might also indicate a preference toward certain TCR gene segments when binding our target peptide (Davis and Bjorkman, 1988). We observe that non-binding rules in general exhibit a far lower density of rules, a sign that they are far less strict than the binding rules. Moreover, many non-binding rules seem to fall in the ‘Other’ region, which may not have any biological significance. This is somewhat expected: the non-binding samples of our dataset (Section 3.2) are randomly sampled from a huge and heterogeneous pool of unspecific TCRs, and therefore may not be characterized by any ‘non-binding pattern’. Of course, there could be other reasons for these biased results that should be investigated, such as TITAN picking up spurious correlations in the dataset.

#### 3.5.2 Visual interpretation of an anchor

**Fig. 7.**
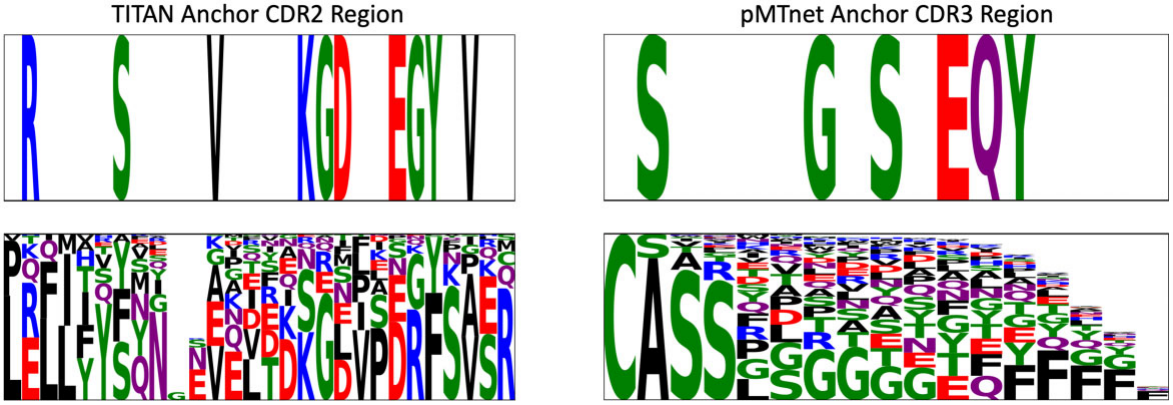
Binding anchor rule examples from TITAN (left) and pMTnet (right). Top row represents the anchor rule, while the bottom row shows the sequence logo of the whole binding split. For TITAN, we focus on the CDR2 region, where many rules fall. pMTnet uses only the CDR3 sequence as input, so gives more specific results for this region

After the preliminary analysis, a user can visually inspect the generated anchor rules, as explained in Section 2.4.1. As a demonstrative example, in Figure 7, we show two binding rules, one from TITAN and one from pMTnet. Each rule (top row) is compared to the sequence logo of the same region (bottom row), which reports the amino-acid frequencies in the binding split. Note that DECODE does *not* simply detect the most frequent amino acids, which would be a trivial task, but instead identifies rules that explain the prediction of TITAN and pMTnet for a particular cluster of samples. Applying DECODE to two different models let us exploit their specific advantages. TITAN uses the whole TCR variable sequence as input and can therefore inform on preferential V or J segment use. Indeed, the anchor shown in Fig. 7 directly implies a use of the TRBV19 segment, indicating that TCRs with this gene segment have a high likelihood of binding the KLGGALQAK-A*03:01 complex we examine. This is a non-trivial finding, as the use of TRBV19 is not higher in the KLGGALQAK binders listed in VDJdb compared to the whole VDJ database. While associations of TRBV segments and epitopes or MHCs are not uncommon, this specific interaction may represent a new biological finding. Nevertheless, experimental validation would be needed to confirm this result. pMTnet on the other hand has only the CDR3 region as input and therefore provides more detailed insight into the features important in this region. The example shown in Figure 2.4.1 is typical for the rules we observe (see Supplementary Material for all anchors), with mainly polar amino acids across the whole CDR3 sequence being of high importance.

## 4 Discussion

In this article, we introduce DECODE, an easy-to-use and customizable pipeline to interpret *any* model for TCR-binding prediction in a faithful and global way. Faithfulness is achieved by leveraging an established high-precision interpretable method, Anchors. Globality is attained by obtaining representative samples of the dataset via *clustering*, and finally applying Anchors to each of them. We demonstrate how easy it is to extract and understand the binding rules by DECODE-ing TITAN and pMTnet, two recently published TCR-binding prediction models.

DECODE is easy to setup thanks to a customizable json configuration file that the user can modify to select the best hyperparameters for their dataset. User-friendliness is further enabled by a range of metrics and visualization tools to help the user in the selection of the best clustering method and the best Anchors parameters, and in the understanding of the (non-)binding rules.

We note that our pipeline can be applied to both good performing and poor performing models. In the former case, the higher the performance of a model, the more likely the extracted rules will have a biological meaning. In the latter case, if the model does not achieve a good accuracy, DECODE may be able to highlight the weaknesses of the model and guide future improvements.

In the future, we plan to expand the selection of clustering algorithms and visualization tools and enable an interactive exploration of the binding rules. Moreover, we aim to further improve the user-friendliness in at least two ways: first, by further simplifying the customization of the configuration file; and, secondly, by providing a framework to guide the user in the selection of the best hyperparameters [e.g. simple grid search, or Bayesian Optimization (Hutter *et al.*, 2011)].

We believe that our work will be able to support the immunology research community in understanding the intricate mechanisms underlying TCR binding, and ultimately contribute to the development of novel and safer immunotherapies.

## Funding

This work was supported by the European Union’s Horizon 2020 Research And Innovation Programme under the Marie Sklodowska-Curie program [Grant Agreement No. 813545] and H2020- ICT-2018-2 program [Grant Agreement No. 826121].

*Conflict of Interest*: none declared.

## Supplementary Material

btac257_Supplementary_Data
