# Supplementary material for: DECODE: a computational pipeline to discover T cell receptor binding rules
Source: Bioinformatics. 2022 Jun 27;38(Suppl 1):i246–54. doi: 10.1093/bioinformatics/btac257 (PMC9235487; doi:10.1093/bioinformatics/btac257)
Supplement: btac257_Supplementary_Data [file btac257_supplementary_data.zip › btac257-suppl_data/Nguyen.100.sup.1.pdf]

Supplementary Material to "DECODE: a computational pipeline to discover T-cell receptor binding rules"

Iliana Papadopoulou, An-phi Nguyen, Anna Weber and María Rodríguez Martínez

S1 More detailed description of anchors metrics

In the following, we describe in more details how to compute the anchors metrics at the different levels, by identifying at each level what we are considering true positives, true negatives, false positives and false negatives.

Example setup As in the main text, we use Figure S1 as a visual aid. Blue stars represent samples predicted as binding by the model we want to explain. Red crosses are samples predicted as non-binding. The thick continuous line is the decision boundary of the model. We can identify 3 clusters for the binding samples, and 2 for the non-binding ones. As an example, we graphically visualize the three levels of metrics applied to *non-binding* anchors. The black crosses denote the medoids of the (non-binding) clusters that we are considering. Dotted circles denote the boundary decisions of the computed anchors. Red rectangles highlight the true positives.

Cluster (CL) level metrics At this level, we are interested in knowing if the (anchor computed on a) medoid of a cluster is able to identify the samples in its own cluster.

- The black cross denotes the medoid we are currently considering.
- The true positives (red rectangle) are the red crosses belonging to the same cluster of the black cross, and located inside the dotted circle. There are 5 true positives: the black cross itself, and the 4 closest red crosses.
- There is a single false negative: the top-most red cross which belongs to the same cluster but is not included in the dotted circle.
- There are 3 false positives: the blue star within the dotted circle, and two red crosses belonging to the other non-binding cluster, but also located within the dotted circle.
- All other points are true negatives.

Cluster-Split (CS) level metrics At this level, we are interested in knowing if the (anchor computed on a) medoid of a cluster is able to identify the samples belonging to the same split.

- The black cross denotes the medoid we are currently considering.
- The true positives (red rectangles) are all the crosses located within the dotted circle.
- The false negatives are the 3 red crosses outside of the dotted circle.
- There is 1 false positive: the blue star within the dotted circle.
- All other points are true negatives.

Split (SP) level metrics At this level, we are interested in knowing if (anchors computed on) all medoids of a split are collectively able to identify the samples belonging to the same split.

- The 2 black crosses denotes the 2 medoids that we are jointly considering.
- The true positives (red rectangles) are all the crosses located within any of the 2 dotted circles.
- There is a single false negative: the top-most red cross outside of the dotted circle.
- There is 1 false positive: the blue star within one of the dotted circles.
- All other points are true negatives.

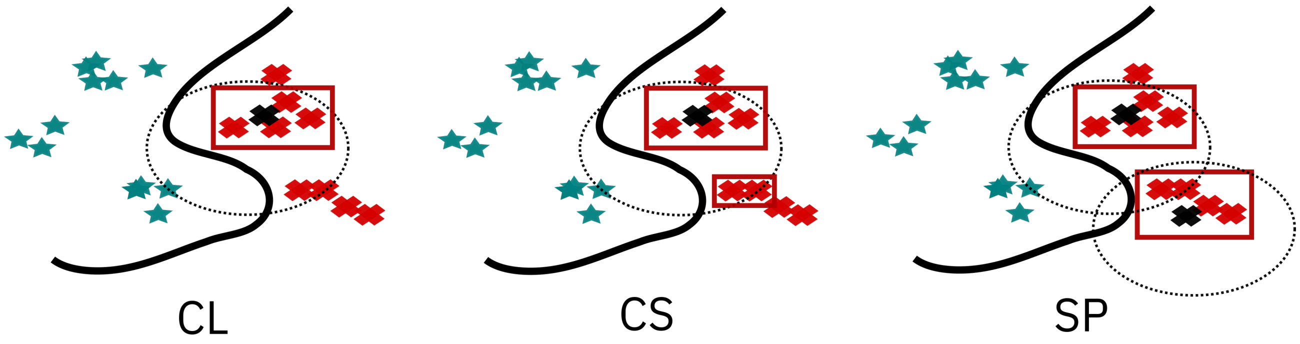

Fig. S1. Cluster metrics: graphical visualization.

S2 Clustering evaluation of the non-binding split.

We report clustering quantitative and qualitative evaluations for the non-binding split. We note that similarly to the binding split, overall the top performing method is `AgglomerativeClustering` with 20 clusters. The best number of clusters for `K-Medoids` is 5, while for `OPTICS` is 38. We not that for all the methods, visually separate groups are clustered together. This is an agreement with our findings that the generated non-binding rules are less precise, but more covering.

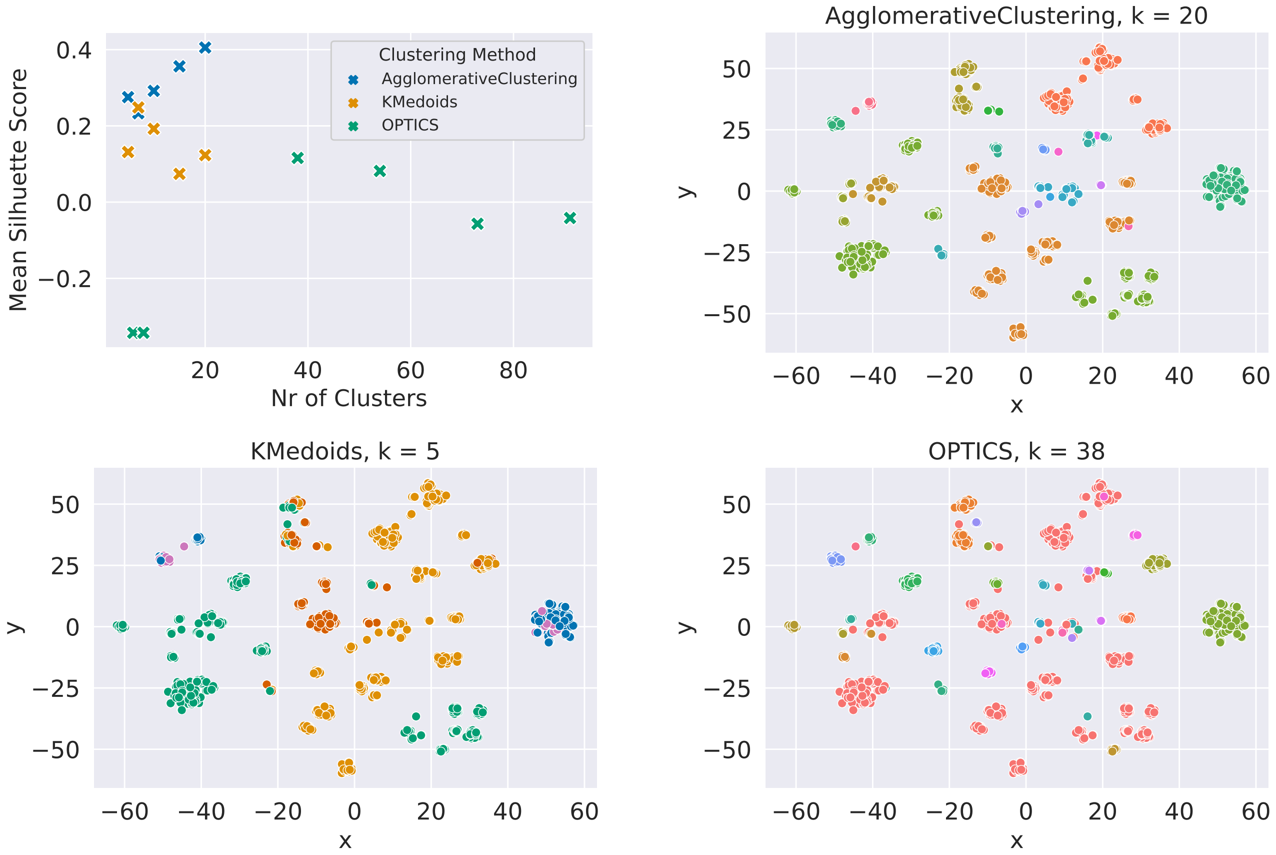

Fig. S2. Comparison of clustering algorithms on the non-binding split. Top-left shows the mean Silhouette score s for all three different clustering methods and a number of different numbers of clusters. `AgglomerativeClustering` achieves high scores for all numbers of clusters, with higher scores for higher numbers of clusters and a maximum of $s = 0.41$ for $k = 20$. The other plots show `t-SNE` plots, where samples are color-coded according to their cluster assignment. The plots were generated for the number of clusters resulting in the highest Silhouette score for each clustering method respectively.

S3 Effect of clustering parameters on anchors performance

We carry out an ablation study to understand the effect of clustering hyperparameters on the anchors performance. For this study, we choose to fix the clustering algorithm (`AgglomerativeClustering`), while we vary the number of clusters (from 5 to 50) and the distance function (Levenshtein vs. BLOSUM-weighted edit distance).

We show our results in Figure S3. As expected, the recall/coverage of anchor rules improves with the number of clusters used. Precision at the cluster level remains largely unchanged, indicating that the number of cluster does not have any effect on the ability of an anchor rule to be applicable to its own cluster. This may be attributed to the fact that the `Anchor` algorithm is designed to be highly precise *in its neighborhood*. Both the cluster-split level and the split level metrics unsurprisingly show a decrease in precision: the more clusters we have, the smaller each cluster becomes. Consequently, the anchor rules will become too specific to their own cluster, and consequently incapable to recognize the whole split. Interestingly, there does not seem to be any appreciable difference in using differently weighted versions of edit distances.

We further analyze how does the completeness of the set of anchor rules vary with respect to the clustering parameters (Figure S4). Again as expected, the higher is the number of clusters, the more complete the set of rules is (i.e. the number of samples that do not fulfill any anchor goes to zero). However, the price to pay for completeness is an increased overlap, as indicated by the fact that with 50 clusters, all samples fulfill more than one anchor rule.

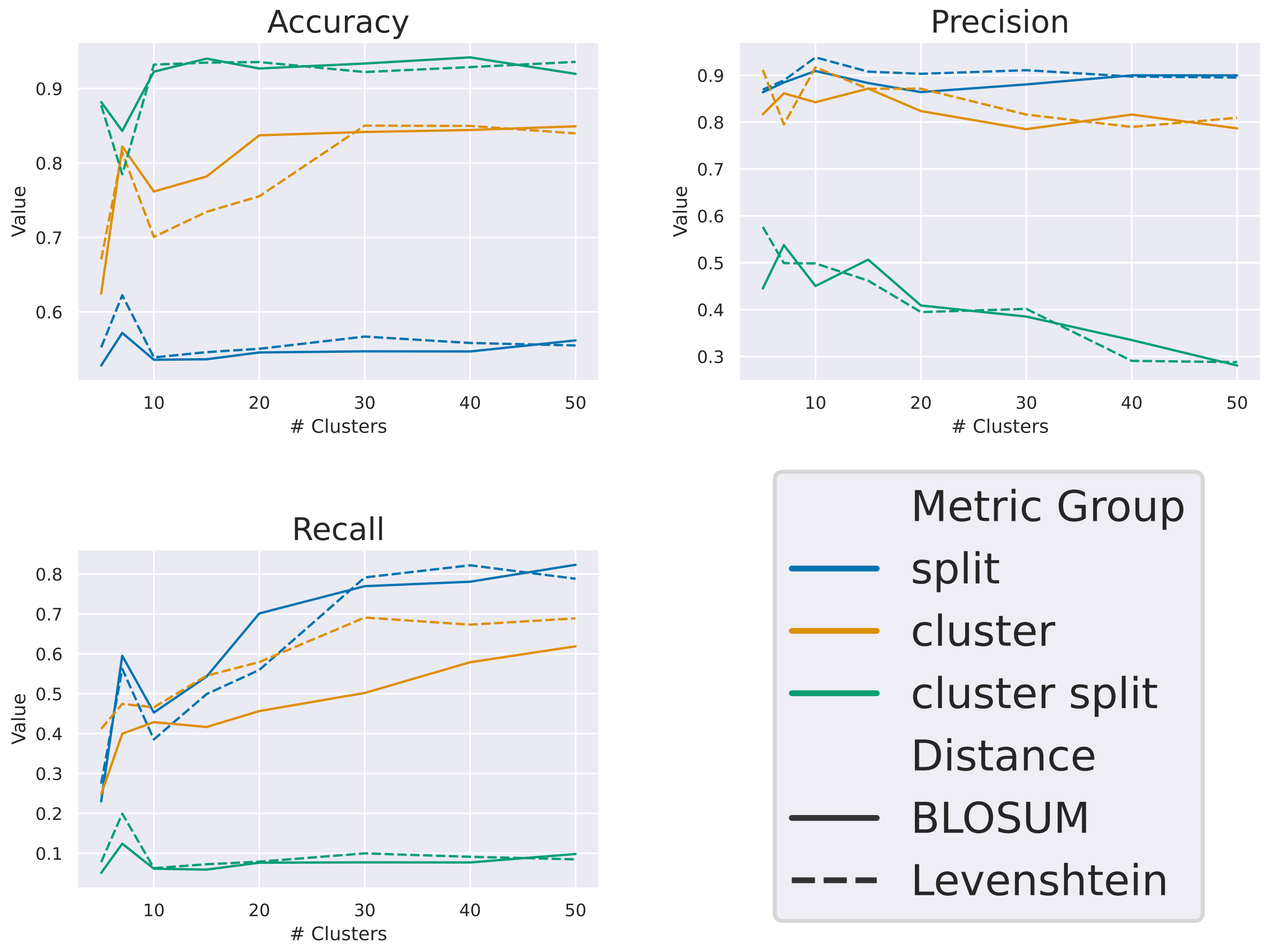

Fig. S3. Analysis of the effect of clustering parameters on anchors metrics. We study the effect of different number of clusters (from 5 to 50). We further study the effect of two different distances: a BLOSUM-weighted distance (solid line) and Levenshtein distance (dashed line). We report the 3 different metrics: accuracy (top-left), precision (top-right), and recall (bottom-left). The colors indicate the 3 different levels at which these metrics can be computed: cluster level (green), cluster-split level (orange) and split level (blue).

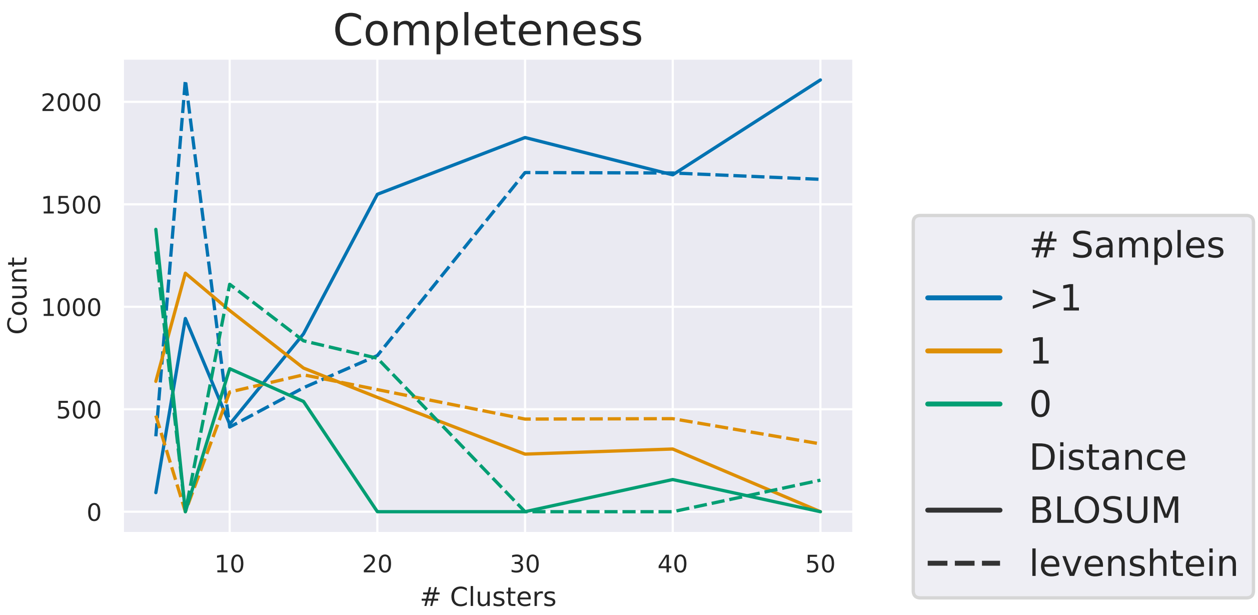

Fig. S4. Analysis of the effect of clustering parameters on the completeness of anchors. We study the effect of different number of clusters (from 5 to 50). We further study the effect of two different distances: a BLOSUM-weighted distance (solid line) and Levenshtein distance (dashed line). For each parameter setting, we count the number of samples that fulfill zero anchor rules (green), exactly one anchor rule (orange), and more than one rule (blue).
